# Nationwide Surveillance for Pathogenic Microorganisms in Groundwater near Carcass Burials Constructed in South Korea in 2010

**DOI:** 10.3390/ijerph10127126

**Published:** 2013-12-12

**Authors:** Ha Kyung Joung, Sang Ha Han, Su-Jung Park, Weon-Hwa Jheong, Tae Seok Ahn, Joong-Bok Lee, Yong-Seok Jeong, Kyung Lib Jang, Gyu-Cheol Lee, Ok-Jae Rhee, Jeong-Woong Park, Soon Young Paik

**Affiliations:** 1 Department of Microbiology, College of Medicine, the Catholic University of Korea, 505 Banpo-dong Seocho-gu, Seoul 137-701, Korea; E-Mails: immalra@naver.com (H.K.J.); lllls3640@gmail.com (S.H.H.); 2 Environmental Infrastructure Research Department, National Institute of Environmental Research, Incheon 404-170, Korea; E-Mails: psj12@korea,kr (S.-J.P.); purify@korea.kr (W.-H.J.); 3 Department of Environmental Science, Kangwon National University, Chuncheon-si, Gangwon-do 200-701, Korea; E-Mail: ahnts@kangwon.ac.kr; 4 Department of Infectious Disease, College of Veterinary Medicine, Konkuk University, Gwangiin-gu, Seoul 143-701, Korea; E-Mail: virus@konkuk.ac.kr; 5 Department of Biology, College of Sciences, Kyung Hee University, 26, Kyungheedaero, Dongdaemun-gu, Seoul 130-701, Korea; E-Mail: ysjeong@khu.ac.kr; 6 Department of Microbiology, College of Natural Sciences, Pusan National University, Busan 609-735, Korea; E-Mail: kljang@pusan.ac.kr; 7 Water Analysis and Research Center, Korea Institute of Water and Environment, Korea Water Resources Corp., Daejeon 306-711, Korea; E-Mail: gclee@kwater.or.kr; 8 DK EcoV Environmental Microbiology Lab, Biotechnology Business Incubating Center, Dankook University, Chungnam 330-714, Korea; E-Mail: ojrh22@nate.com; 9 Sanigen Co. Ltd., Juan-dong, Gwacheon 427-070, Korea; E-Mail: withgaia@sanigen.kr

**Keywords:** carcass burial, groundwater, pathogenic microorganism, public health

## Abstract

Widespread outbreaks of foot-and-mouth disease and avian influenza occurred in South Korea during 2010. In response to the culling of many animals to attenuate the spread of disease, South Korea used mass burial sites to dispose of the large number of carcasses; consequently, concerns about groundwater contamination by leachate from these burial sites are increasing. Groundwater is one of the main sources of drinking water, and its cleanliness is directly related to public health. Thus, this study aimed to evaluate the safety of groundwater around the burial sites (total of 600 sites). A total of 1,200 groundwater samples were collected though the country, and microbial analysis was conducted during two time periods: during the spring (*n* = 600; April to June 2012) and after rainfall (*n* = 600; August to October, 2012; fall). Fecal coliform and *Escherichia coli* were detected in 173 (14.4%) and 85 (7.1%) of the 1,200 samples, respectively. *Salmonella* spp. and *Shigella* spp. each were detected only once (0.083%). *Clostridium perfringens* was detected from 7 groundwater samples (0.583%), and *E*. *coli* O157:H7 was not detected. With respect to norovirus, only the GII type was detected from six groundwater samples (0.5%), and enterovirus was detected in 15 groundwater samples (1.25%). The frequency of *E*. *coli* that we detected was lower than that found in previous studies conducted in South Korea, but we detected higher frequency of fecal coliform than that observed in a previous report. The contamination frequencies of *Salmonella* spp. and *Shigella* spp. were very low, but *C*. *perfringens*, which could be an indicator of fecal pollution, was detected in seven regions. Overall, the results of the present study indicate a low possibility of contamination from burial sites. However, consistent monitoring is required to prevent microbial contamination of groundwater near the burial sites.

## 1. Introduction

Groundwater comprises a large portion of drinking water and industrial or household water in many countries [1]; in South Korea, 11% of the total used water is supplied by groundwater [2]. Because groundwater is shared within communities, pathogen-contaminated groundwater can cause large-scale outbreaks of waterborne diseases. Waterborne disease outbreaks due to contaminated drinking water have been frequently reported around the world [3]. During the period between 2007–2008, a total of 48 waterborne disease outbreaks were reported in 24 states in the United States and Puerto Rico, including 29 outbreaks in 2007 and 19 outbreaks in 2008 [4]. Among them, 36 outbreaks were associated with drinking water, resulting in 4,128 ill patients and three deaths and five outbreaks were associated with bacterial agents and viral agents [4]. Groundwater was implicated in these outbreaks [4].

Waterborne diseases can be caused by the presence of various microorganisms, especially bacteria and viruses, in groundwater. Pathogenic bacteria include *Salmonella* spp., *Shigella* spp., *Escherichia coli*, and *Campylobacter jejuni* [5]. In addition, *Clostridium* is often the cause of waterborne disease when groundwater is contaminated with fecal material [6]. The prominent pathogenic viruses, which include norovirus (NV), enterovirus, rotavirus, and hepatitis A and E, may be associated with waterborne disease [5]. Generally, most symptoms of waterborne disease begin with acute gastrointestinal illness, resulting in stomach cramps, vomiting, nausea, fever, and diarrhea [6,7]. Depending on the individual, the symptoms can be severe or even fatal.

There are various factors that result in the contamination of groundwater with microorganisms; among them, the disposal of carcasses is a current concern. Methods for the disposal of carcasses include burial, burning, incineration, composting, and rendering [8]. Burial and burning are methods commonly used worldwide for disposal; burial, in particular, is widely used for mass carcass disposal [8]. If leachate from carcass burial permeates the soil and reaches groundwater, this process could contaminate the groundwater with pathogens. Further, as leachate carries chemical compounds, these compounds can be a source of nutrients for the growth and multiplication of bacteria [9]. According to Ho *et al*. the burial of foot-and-mouth disease (FMD)-infected pigs was related to enterovirus infection in Taiwan [10]. In this case, more than 3,000 people were infected with hand-foot-and-mouth disease, and 85 children died (caused by 67 subtypes of enteroviruses) [10]. Even though this large outbreak from contaminated groundwater has been documented, few studies have been performed to examine soil and groundwater contamination due to leachate from carcass burial [9]. To date, the studies of groundwater near carcass burial sites investigated chloride, nutrients, and fecal pathogens, with a limited the number of samples, and mainly focused on poultry [11]. According to Ritter *et al*. a high ammonium concentration was detected in the groundwater around a bird carcass site, and the concentrations of fecal coliform (FC) and fecal streptococci were low [12]. Yuan *et al*. also detected a high concentration of ammonium and other substrates (steroid hormones, veterinary antibiotics, carbon, nutrients, and solid matter) from the land burial of cattle carcasses [11], and the authors suggested that the leachate could have been the source of microbial contamination in the groundwater [11].

As widespread infectious diseases in animals, FMD and avian influenza (AI) resulted in culling of infected animals and disposal by burial [9]. FMD is caused by the highly infectious foot-and-mouth disease virus (FMDV). Ungulate mammals, such as cow, swine, goat, and sheep, are susceptible to FMD [13]. AI is an influenza virus infecting birds, such as ducks and geese, and some of these AI viruses can cross species to infect even humans [14]. In South Korea, burial of carcasses was performed regardless of species, according to “The regulation about water quality standard and examination of drinking water” [15] from the Ministry of Environment of South Korea. The burial sites in South Korea for FMD- and AI- infected animals must be constructed more than 1 m from the surface of groundwater and more than 30 m from rivers, streams, catchments, and residential areas. The recommended size of the burial pit depends on the amount of carcasses, but it should be no more than 4–5 m wide and 5 m deep. The slope of the bottom should be more than 2% grade. Mixed clay including clay minerals (*i*.*e*., bentonite) is used to cover the bottom and sides of the burial pit. Then, the mixed clay is covered with impermeable duplex vinyl and soil. The carcasses are buried and covered with soil and quicklime. The burial mound is covered with quicklime and vinyl. A drainpipe is installed at the bottom of the pit to prevent leachate from flowing out [13]. In 2010, a large FMD outbreak occurred in East Asia (South Korea, Hong Kong, Mongolia, China, Japan, and Russia) [14], and a total of 3,535,782 animals were slaughtered [9]. In addition, 6,470,000 birds were culled due to the presence of AI in the flocks in 2010 [9]. As a result, more than 4,599 burial pits were made in 2010 [9]. If the leachate from the burial sites contaminated groundwater, the contaminated water could expose the population to pathogens and lead to potential outbreaks of infections. Therefore, the numbers of pathogenic microorganisms in the groundwater must be monitored regularly. 

Due to its importance as a source of drinking water, many studies have been conducted to investigate the quality of groundwater in South Korea [16,17,18,19]. Lee *et al*. performed groundwater monitoring with a total of 600 samples during the summer (June–August) and winter (October–December) in 2008 from seven regions of South Korea [16]. The authors detected NV in a total of 117 samples (117/600, 19.5%). Among them, 65 of the positive samples were detected in the summer (65/300, 21.7%), and 52 positive samples were detected in the fall (52/300, 17.3%) [16]. Choi *et al*. also tested a total of 197 groundwater samples during April 2005 to September 2005 in Busan, South Korea, and the authors found that 71 regions were contaminated with total coliforms, while fecal coliforms were detected in 22 regions [17]. *Salmonella* and *Shigella* were not detected anywhere [17]. Another study was performed by Cheong *et al*. from August 2007 to May 2008 in Gyeonggi-do, South Korea, and they reported that five out of 29 groundwater samples were contaminated with enterovirus [18]. Also, total coliforms, fecal coliforms, and enterococci were detected in eight samples (8/29, 28%), six samples (6/29, 21%), and four samples (4/29, 14%), respectively [18]. In 2010, Lee *et al*. investigated a total of 1,090 groundwater samples, which were used in food catering facilities in 2010 from eight provinces in South Korea, and detected NV in seven samples, representing 0.64% of the total tested samples [19].

Although many studies examining microbial groundwater quality were performed on a national scale, there has been no nationwide study to evaluate the safety of groundwater after the mass burials in South Korea. Therefore, this study was necessary to insure the safety of people who use the groundwater as drinking water. This study tested 1,200 groundwater samples collected around a total of 600 burial sites constructed in 2010 throughout four provinces to investigate microbial quality, particularly pathogenic microorganisms, of the groundwater. 

## 2. Materials and Methods

### 2.1. Sampling Sites

**Figure 1:**
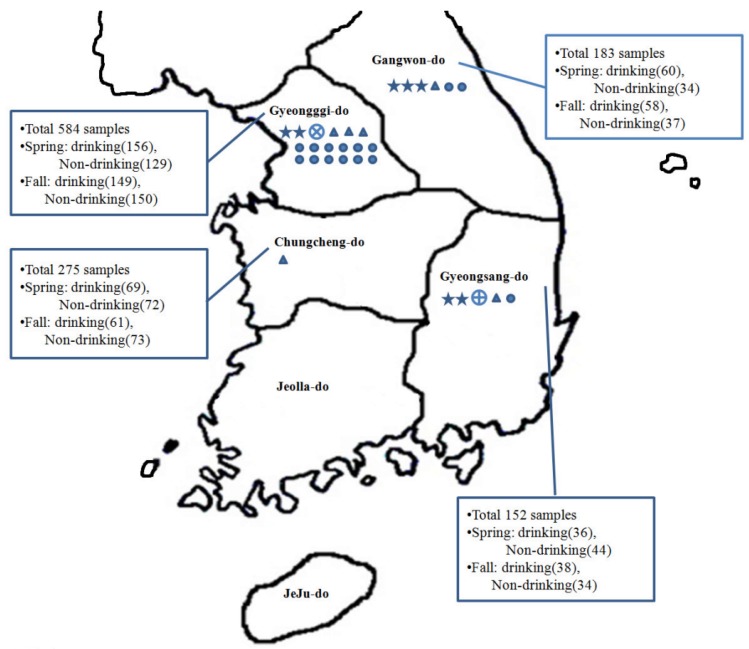
Map showing groundwater sampling locations and pathogen distribution. Samples were collected from a total of 1,200 sites. By region, 584 samples were from Gyeonggi-do, 183 were from Gangwon-do, 275 were from Chungcheng-do, and 152 were from Gyeongsang-do. The distribution of pathogenic microorganisms is marked with the following symbols: ✩*Clostridium perfringens*, ⨁*Salmonella*, ⨂*Shigella*, ∆norovirus, and ○enterovirus. Groundwater from Gyeonggi-do was found to be most contaminated with pathogenic microorganisms, followed by that from Gangwon-do, Gyeongsang-do, and Chungcheng-do.

A total of 1,200 sampling sites near a total of 600 burial sites were selected by the National Institute of Environmental Research (≤1,000 m from burial). The selection of sampling sites was based on proximity to burial sites and potential groundwater contamination due to carcass burials during the FMD and AI outbreaks in 2010. The species of culled animals were pigs, cattle, goats, and deer for FMD and chickens for AI. The number of carcasses in each pit was between 1 and 44,706, and the regions with burial pits were Gyeongsang-do, Gyeonggi-do, Gangwon-do, and Chungcheng-do in South Korea (Figure 1). Samples (*n* = 1,200; drinking and non-drinking groundwater) were collected over the following two time periods to investigate seasonal differences: earlier in the thawing season (*n* = 600, April–June 2012; spring) and after rainfall (*n* = 600, August–October 2012; fall). During the spring, frozen soil is permeable to melting snow and ice, and leachate from carcass burial sites could affect the groundwater. In summer, the heavy rainfall in this area causes too many factors that could affect groundwater. Therefore, spring and fall were selected as the sampling periods. Burial sites were far from each other, hence each sampling from each groundwater was performed from one burial site.

There were 321 (53.5%) drinking water samples collected in the spring, and 306 (51%) collected in the fall. The other samples were collected from non-drinking water sites.

### 2.2. Groundwater Filtering

According to the standard procedure by Fout *et al*. and Parshionikar *et al*. [20,21], 20 gal (76 L) of groundwater were filtered through an electropositive filter (Virsorb^®^ 1-MDS; ZetaPorVirosorp, Cuno, Meriden, CO, USA) and used for detection of NV and pan-enterovirus. For detection of pathogenic bacteria, 3 L of water were filtered.

### 2.3. Water Quality Analysis

The following characteristics of the groundwater samples were measured to investigate water quality: pH (Lutron pH-208, Electronic Enterprise Ltd., Taipei, Taiwan), turbidity (Hanna HI93703, Chelmsford, England), residual chlorine concentration (Hanna HI9570, Chelmsford, England), and temperature (Lutron pH-208, Hsin-Chu, Taiwan).

### 2.4. Bacterial Analysis

Detection of microbial contaminants in groundwater samples was performed using modified procedures in the “Environmental Standard Methods for Drinking Water” [22] from the Ministry of Environment in South Korea and in the “Food Code 2008” [23] from the Ministry of Food and Drug Safety in South Korea.

FC and *E*. *coli* were primarily detected using the Colilert^®^ kit (IDEXX, Westbrook, Maine, USA). The concentration of the bacteria was determined based on the number of color-changed squares, and the number was converted to the most probable number (MPN) by the MPN method using QuantiTray^®^/2000.

To detect *E*. *coli* O157:H7, 250 mL of groundwater were filtered through a membrane filter (0.4 μm pore size, 47 mm diameter; Millipore Corp., Billerica, MA, USA). The membrane filters were then placed in modified *Escherichia coli* broth (BD, Sparks, MD, USA) for enrichment and incubated at 37 °C for 24 h. The samples showing turbidity were then sub-cultured onto MacConkey sorbitol agar (BD) plates containing cerfixime (0.05 μg/L, Lab M, Bury, UK) and potassium tellurite (2.5 μg/mL), and then the plates were incubated for 18–24 h at 37 °C. Prospective colonies were plated onto Eosin Methylene Blue (BD) agar plates for confirmation, and positive *E*. *coli* O157:H7 colonies were isolated and enriched on nutrient agar (NA; BD) plates. 

For *Salmonella* and *Shigella* detection, water samples were filtered as mentioned above, and the membrane filters were placed in selenite broth (BD) for enrichment. If the color of selenite broth changed to red, indicating bacterial growth, the enriched samples were spread onto a bismuth sulfate agar (BD) plate for *Salmonella* and onto a xylose lysine deoxycholate (BD) agar plate for *Shigella*. After incubation for 24 h, positive colonies were isolated and inoculated onto NA plates.

To isolate *C*. *perfringens*, 200 mL groundwater samples were heated at 75 °C for 15 min and filtered. The membrane filters were placed on a tryptone sulfite cycloserine agar plate (Lab M), and then plates were incubated at 37 °C anaerobic conditions for 24 ± 2 h. After incubation, if black or grey colonies appeared, five colonies were inoculated onto NA plates and incubated in aerobic and anaerobic conditions. Only colonies capable of growing anaerobically were confirmed as *C*. *perfringens* and isolated for inoculation onto NA plates. The presumed positive colonies of pathogenic microorganisms were sent to Macrogen (Seoul, South Korea) for further identification.

### 2.5. Viral Analysis

After filtration with a 1-MDS filter, approximately 20 mL of water was eluted, and viral RNA was extracted from the eluate using the QIAamp viral RNA minikit (Qiagen, Hilden, Germany) according to the manufacturer’s instructions. 

For NV and enterovirus detection, RT-PCR was performed with a Verso 1-step RT-PCR Hot-Start Kit (ABgene, Foster, California, USA), and semi-nested PCR was conducted using a 2X EmeraldAmp PCR Master Mix (TaKaRa, Shiga, Japan). The specific primer sets for PCR analysis for each virus are described in Table 1. As positive controls for NV GI and NV GII, standard positive controls were used, and they were provided by Waterborne Virus Bank of Korea [24]. The controls, made by Lee *et al.*, allow for reliable diagnosis of NV by RT-PCR and are designed to be size distinguishable [25]. An S1000^TM^ Thermal Cycler (BIO-RAD, Singapore) was used for PCR, and the RT-PCR thermocycling steps for NV were the following: first step, 50 °C for 15 min and 95 °C for 15 min; amplification cycle (repeated 35 times), 94 °C for 30 s, 60 °C for 45 s, 72 °C for 1 min and 30 s; and a final step, 72 °C for 7 min. The components for RT-PCR were viral RNA 5 μL, 2X 1-step PCR Hot-Start Master Mix 9.5 μL, RT enhancer 1 μL, Verso RT enzymix 0.5 μL, and 2 μL of each primer set. The components for nested PCR were EmeraldAmp PCR Master Mix (2X Premix) 25 μL, forward primer 2 μL, reverse primer 2 μL, distilled water 19 μL, and RT-PCR product 2 μL. The nested PCR thermocycling steps were the following: initial step, 94 °C for 5 min; amplification cycle (repeated 25 times), 94 °C for 30 s, 55 °C for 30 s, 72 °C for 1 min and 30 s; and final extension step, 72 °C for 7 min. Nested PCR products were stored at 4 °C. The PCR products were visualized upon conducting gel electrophoresis. Positive samples were identified as those having a band of expected size (NV GI: 313 bp, NV GII: 310 bp).

**Table 1:** Primer sets for detection of viruses by PCR.

Genotype	Primer	Sequence (5'→3')	Product size (bp)	Reference
Norovirus GI	GI-F1M ^a^	CTGCCCGAATTYGTAAATGATGAT	313	[26]
	GI-F2 ^b^	ATGATGATGGCGTCTAAGGACGC		
	GI-R1M ^a,b^	CCAACCCARCCATTRTACATYTG		
Norovirus GII	GII-F1M ^a^	GGGAGGGCGATCGCAATCT	310	
	GII-F3M ^b^	TTGTGAATGAAGATGGCGTCGART		
	GII-R1M ^a,b^	CCRCCIGCATRICCRTTRTACAT		
Enterovirus	EV1^ a,b^	TCC GGC CCC TGA ATG CGG CT	105	[27]
	EV2^ a^	TGT CAC CAT AAG CAG CC		
	EV3^ b^	CCC AAA GTA GTC GGT TCC CC		

Notes: **^a^** Primer sets for RT-PCR; **^b^** Primer sets for semi-nested PCR.

The components for enterovirus PCR were similar to NV PCR, with the exception of the primer sets. The RT-PCR steps for enterovirus detection were the following: first step (same as for NV detection), 50 °C for 15 min and 95 °C for 15 min; amplification cycle (repeated 30 times), denaturation at 94 °C for 30 s, annealing at 60 °C for 45 s, and elongation at 72 °C for 1 min; final step, 72 °C for 10 min. After the RT-PCR, semi-nested PCR was performed using the following steps: amplification cycle (repeated 30 times), 98 °C for 10 s, 60 °C for 45 s, and 72 °C for 1 min; and final step, 72 °C for 10 min. The PCR products were separated on a 1.5% agarose gel, stained with ethidium bromide, and visualized under UV light. The positive PCR products appeared as 105 bp size bands. The products were extracted from the agarose gel with a HiYield^TM^ Gel/PCR DNA Fragments Extraction Kit (Real Genomics^TM^, Real Biotech Corp., Taipei, Taiwan), and the sequences were analyzed by Cosmogenetech (Seoul, South Korea). Each sequence was compared with a GenBank nucleotide database using the BLAST program (NCBI) [28].

### 2.6. Phylogenetic Analysis

Phylogenetic analysis was performed with DNAStar version 5.07 software. The DNA sequence alignments and phylogenetic tree were conducted using the neighbor-joining and CLUSTAL W methods.

### 2.7. Nucleotide Sequence Accession Numbers

The sequences of NV isolated from groundwater samples were submitted to GenBank of NCBI [28]. The deposited accession numbers are KC800916, KF015248, KF015246, KF015250, KF015251, and KF015252.

### 2.8. Statistical Analysis

Data are expressed as mean value of frequency ± SE. The correlation both within microorganism type and between microorganism type and physicochemical data (temperature, pH, turbidity, distance from burial, the amount of carcasses, and depth of the groundwater) was determined with *t*-test, correlation, and Chi-square, using SPSS statistical software (Version 12, SPSS, Inc., Chicago, IL, USA).

## 3. Results

### 3.1. Measurement of the Temperature, Turbidity, and pH

The temperatures in each season were 15.5 °C ± 1.6 (range, 9.5–25.0 °C) in the spring and 16.5 °C ± 2.0 (range, 16.4–24.4 °C) in the fall. Similar temperatures were also measured in drinking water (15.5 °C ± 1.6 in the spring and 16.4 °C ± 1.8 in the fall) and non-drinking water (15.5 °C ± 1.7 in the spring and 16.6 °C ± 2.1 in the fall) (Table 2).

**Table 2:** Average of temperature, turbidity, and pH.

	Temperature ( °C)		Turbidity (NTU)		pH (pH)	
	Spring	Fall	Spring	Fall	Spring	Fall
Drinking	15.5 ± 1.6	16.4 ± 1.8	0.1 ± 0.4	0.3 ± 0.3	7.0 ± 0.6	7.0 ± 0.4
Non-drinking	15.5 ± 1.7	16.6 ± 2.1	0.2 ± 1.9	0.4 ± 0.4	7.0 ± 0.4	7.0 ± 0.4
Total	15.5 ± 1.6	16.5 ± 2.0	0.1 ± 1.4	0.4 ± 0.4	7.0 ± 0.5	7.0 ± 0.4

In the fall survey, turbidity was somewhat higher than that in the spring. The average turbidity was 0.1 ± 0.4 nephelometric turbidity unit (NTU) in the spring and 0.4 NTU ± 0.4 in the fall. The average pH in spring and fall was 7.0 ± 0.5 and 7.0 ± 0.4, respectively. The average pH in drinking water was 7.0 ± 0.6 in the spring and 7.0 ± 0.4 in the fall. Similarly, the average pH in non-drinking water was 7.0 ± 0.4 in both seasons (Table 2).

There was no noticeable deviation in temperature, turbidity, and pH with respect to the distance between burial and sampling sites. To investigate the relationships between chemical data (pH, temperature, turbidity) and each microorganism, statistical analysis was performed; however, reliable statistical data was not obtained, as the detection rates of pathogenic microorganisms were too low. In the analysis of correlation between chemical data and non-pathogenic microorganisms (fecal coliform and *E*. *coli*), there were no significant correlations (*p* ≥ 0.05) (data not shown).

### 3.2. Distance between Burial and Sampling Sites

The detection rates of microorganisms by distance between burial and sampling sites is shown in Table 3. This study focused on the groundwater, which was within a 200 m radius from the burial site. The detection rates of fecal coliform, *E*. *coli*, *C*. *perfringens*, and NV GII within a 0–50 m radius from burial (12.7%, 6.8%, 0.4%, and 0.8%, respectively) were similar to rates within a 51–100 m radius from burial (13.3%, 6.1%, 0.4%, and 0.4%, respectively). Also, the detection rates of the pathogens within a 0–100 m radius (13%, 6.4%, 0.4%, and 0.6%) were similar to rates within a 101–200 m radius (15.1%, 7.3%, 0.7%, and 0.7%).

The statistical analysis of correlation between microorganisms (fecal coliform and *E*. *coli*) and distance between sampling site and burial was conducted, but there were no significant differences between them (*p* ≥ 0.05) (data not shown). Although the analysis of relationships between pathogenic microorganisms and distance was executed, meaningful results were not obtained as there were too few positive samples of pathogenic microorganisms.

### 3.3. Detection of Pathogenic Bacteria

Figure 1 shows the presence of pathogenic bacteria in four burial provinces (“-do”). Detection of bacterial pathogens in this study was a rare event; there were only nine samples from which pathogens were recovered: four in the spring and five in the late summer and fall; four cases in drinking water and five cases in non-drinking water. Among the tested pathogenic bacteria, *C*. *perfringens* was the most prevalent throughout the country, as it was detected in seven regions (0.6%, 7/1200 samples), and it was not detected only in Chungcheng-do. The detection frequency of *C*. *perfringens* was 0.7% (4/600) in the fall and 0.5% (3/600) in the spring. Non-drinking water contained more *C*. *perfringens* than drinking water (non-drinking: 5/1,200 (0.9%); drinking: 2/1,200 (0.3%)).

*Salmonella* was detected in only one drinking water sample from Gyeongsang-do in the fall. *Shigella* was also detected only once in drinking water from Gyeonggi-do during the spring survey. *E*. *coli* O157:H7 was not detected in any of the collected water samples. 

### 3.4. Detection of Fecal Coliforms and E. coli

FCs were detected in 14.4% (173/1200) of the samples. The mean concentration of FC was 87.0 MPN ± 298.8/100 mL. In this survey, the total detection rate of FC in the spring (9.2%, 55/600) was less than that of the fall (19.7%, 118/600). The detection rate of FC in drinking water was somewhat higher (25.4%, 101/627) than that in non-drinking water (12.6%, 72/573), and the FC detection rate in drinking water was higher than that in non-drinking water for both seasons.

**Table 3:** Distance from burial and detection of microorganisms.

Distance from burial site (m)	Number of samples	Fecal coliform	*E*. *coli*	*Clostridium perfringens*	Norovirus GII	Enterovirus	*Salmonella*	*Shigella*
0–50	237	30 (12.7%)	16 (6.8%)	1 (0.4%)	2 (0.8%)	3 (1.3%)	0	1 (0.4%)
51–100	279	37 (13.3%)	17 (6.1%)	1 (0.4%)	1 (0.4%)	4 (1.4%)	0	0
101–200	449	68 (15.1%)	33 (7.3%)	3 (0.7%)	3 (0.7%)	5 (1.1%)	1 (0.2%)	0
201–300	165	20 (12.1%)	6 (3.6%)	1 (0.6%)	0	2 (1.2%)	0	0
301–400	11	2 (18.2%)	1 (9.1%)	0	0	1 (9.1%)	0	0
401–500	41	10 (24.4%)	7 (17.1%)	1 (2.4%)	0	0	0	0
501–600	4	3 (75.0%)	3 (75.0%)	0	0	0	0	0
601–700	4	1 (25.0%)	0	0	0	0	0	0
701–1,000	10	2 (20.0%)	1 (10%)	0	0	0	0	0

**Table 4:** Detection of microorganisms.

	Fecal coliforms		*E*. *coli*		*Clostridium perfringens*		*Salmonella*		*Shigella*		Norovirus GII		Enterovirus	
	Spring	Fall	Spring	Fall	Spring	Fall	Spring	Fall	Spring	Fall	Spring	Fall	Spring	Fall
Drinking	37/321 (11.5%)	64/306 (20.9%)	13/321 (4.0%)	33/306 (10.8%)	1/321 (0.3%)	1/306 (0.3%)	0/321	1/306 (0.3%)	1/321 (0.3%)	0/306	5/321 (1.6%)	0/306	1/321 (0.3%)	8/306 (2.6%)
Non-drinking	18/279 (6.5%)	54/294 (18.4%)	9/279 (3.2%)	30/294 (10.2%)	2/279 (0.7%)	3/394 (1.0%)	0/279	0/294	0/279	0/294	1/279 (0.4%)	0/294	2/279 (0.7%)	4/294 (1.4%)
Total	55/600 (9.2%)	118/600 (19.7%)	22/600 (3.7%)	63/600 (10.5%)	3/600 (0.5%)	4/600 (0.7%)	0/600	1/600 (0.2%)	1/600 (0.2%)	0/600	6/600 (1.0%)	0/600	3/600 (0.5%)	12/600 (2%)

*E*. *coli* was detected in 7.0% (85/1200) of the samples. In the fall, average detection rates of *E*. *coli* in drinking and non-drinking water were 16.7 MPN ± 28.7/100 mL and 11.7 MPN ± 18.1/100 mL, respectively. In the spring, the *E*. *coli* detection rate in non-drinking water (mean, 391.4 MPN ± 853.6/100 mL) was higher than in drinking water (mean, 8.3 MPN ± 9.5/100 mL). 

There were significant relationships between fecal coliform and *E*. *coli* (*p* < 0.0001).The detection rate of *E*. *coli* in the regions tested for fecal coliform was 32.73% in the spring and 45.76% in the fall. Additionally, the detection rate of fecal coliform in the regions tested positive for *E*. *coli* was 81.82% in the spring and 85.71% in the fall. However, there was no correlation between physicochemical data (pH, turbidity, temperature, distance between burial site and groundwater, number of buried animals, and depth of the groundwater) and each microorganism (fecal coliform and *E*. *coli*) (*p* ≥ 0.05) (data not shown).

### 3.5. Detection of Pathogenic Viruses

Detected regions of pathogenic viruses are shown in Figure 1. A total of six groundwater samples were contaminated by NV GII, and NVs were detected only in the spring. NV GI was not detected in either season. Drinking water was more contaminated than non-drinking water with NV (drinking water: five samples (1.6%); non-drinking water: one sample (0.4%)), with Gyeonggi-do groundwater being the region most polluted with NV. 

Based on sequencing data, the most frequently detected genotype was GII.4, and a total of five samples of GII.4 serotypes were detected throughout the nation; genes for the detected serotypes are named *KF015246*, *KF015250*, *KC800916*, *KF015252*, and *KF015251*. The *KF015248* sample could not be analyzed for subtype because only limited sequence data were obtained. The most similar genes among the five samples were *GII/SN02-52-KOR* (accession No. HQ148247) and GII.4/Taoyuan CGMH59-TW (*KC517369*). These two genes had 100% identity with the specific partial sequences compared from the present study. The percentage of identities between the genes most likely homologous and the sequences from detected samples were the following: *KF015246*, *KF015251*, *KF015252*, *KC800916*, and *KF015250* were 98.6%, 98.2%, 98.2%, 97.7%, and 97.7%, respectively. The sequences of the detected NV GII.4 cases were similar to each other. The identity between *KF015251* and *KF015252* was 100%. In addition, the identity between *KF015246* and *KF800916* was 99.1%. *KF015250* also had 98.6% identity with the GII.4 samples, *KF015251*, *KF015252*, *KF015246*, and *KC800916*. However, for *KF015248*, sequence identities were low when compared with other NV GII sequences. The other gene most similar to *KF015248* was *KC800916*, with 43.1% identity; furthermore, from a BLAST search, Bristol-UK (*X76716*) had 42.5% identity.

**Figure 2:**
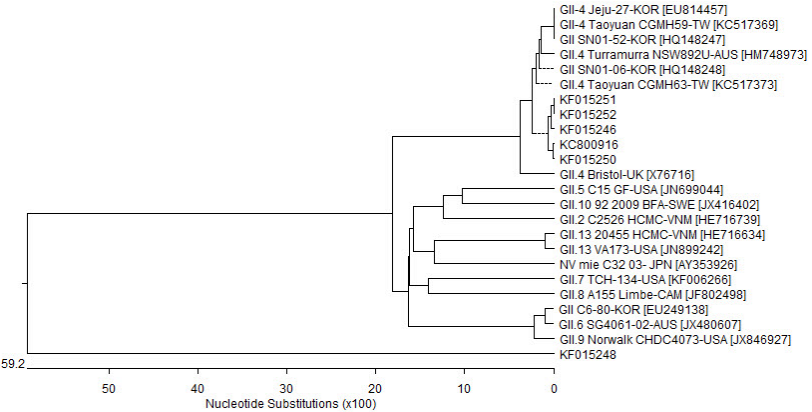
Phylogenetic tree of capsid genes in viruses. The phylogenetic tree was made using the partial sequence of the capsid gene, which was detected in norovirus GII. The detection was conducted by polymerase chain reaction with the norovirus primer set listed in Table 1.

A phylogenetic tree of NV GII gene sequences detected in this study is shown in Figure 2. As mentioned above, *KF015251*, *KF015252*, *KF015246*, *KC800916*, and *KF015250* were closely associated with GII.4 types. However, *KF015248* was not related to any subtype of genes. When the sequence of *KF015248* was compared with a nucleotide database of GenBank, the most similar gene was NV/mie/32/03/JPN (*AY353926*), which was related to NV GII.13. However, based on phylogenetic analysis, the subtype of *KF015248* could not be determined.

Of the 1,200 samples, 15 samples (1.3%) were confirmed positive for pen**-**enterovirus. As shown in Table 4, the number of contaminated groundwater samples with enterovirus was higher in the fall than in the spring. Enterovirus was more frequently detected in drinking water than in non-drinking water. Regionally, Gyeonggi-do had the highest prevalence of enterovirus (12 cases out of 15 total cases).

## 4. Discussion and Conclusions

Many countries use the mass burial method to dispose of large numbers of dead animals. Because leachate from the burial sites could permeate the soil and reach groundwater, a method is needed to monitor the surrounding environment. Moreover, leachate could include various chemical compounds that could provide nutrients to bacteria for their growth and multiplication [9]. Hence, if groundwater is contaminated with leachate, and people use the contaminated water, this could lead to human infection from ingestion of harmful microorganisms. An unfortunate example of this possibility occurred in 1998 with a huge outbreak of enterovirus from contaminated groundwater in Taiwan [10].

There were large-scale outbreaks of FMD and AI in 2010 in Asia, including South Korea, and enormous numbers of slaughtered animals were buried for disposal. This study focused on the groundwater near the carcass burials sites, and we determined that samples contained fecal contamination. We supposed that the origin of contamination was possibly buried carcass intestines or discarded feces from live animals on ranches. Thus, to clarify the origin of fecal contamination, further investigation of the groundwater around ranches and comparison with the results of this study is needed. 

Overall, drinking water samples contained more microorganisms (165/1,200 (26.3%)) than non-drinking water samples (123/1,200 (21.5%)), with the exception of *C*. *perfringens*.Lee *et al*. reported many cases in South Korea of drinking water contaminated with NV, general bacteria, *E*. *coli*, otal coliform, somatic phage, male-specific phage, and pan-enterovirus [16]. The study reported that a total of 117 of 300 groundwater samples contained NV, and 70 of the 117 contaminated water samples were drinking water. Moreover, a total of 11 groundwater samples were contaminated with pan-enterovirus, and 6 of those samples were drinking water [16]. According to the drinking water guideline from the World Health Organization, “*Water entering the distribution system must be microbiologically safe and ideally should also be biological stable*” [29]; however, the results in this study of the quality of drinking water near the burial sites contravenes the guidelines, and the water was also unsuitable according to the regulation of the Ministry of Environment of South Korea.

Overall, detection rates for FC, *E*. *coli*, *Salmonella*, and *Clostridium* were higher in the fall than in the spring. Due to the humidity and high temperature in summer, soil and groundwater have the potential to collect pathogenic bacteria from leachate of burials or other contamination sources such as ranches, and these bacteria could flourish in the groundwater after summer to be detected in the fall.

The pathogenic bacterium most frequently detected was *C*. *perfringens*, and it was detected in a total of seven groundwater samples. Because *C*. *perfringens* has been regarded as an indicator of fecal pollution [30], it is likely that groundwater near the burial sites was contaminated with leachate from carcasses. Efuntoye *et al*. reported that 14 strains of *C*. *perfringens* were present in landfill leachate in Ibadan, Nigeria, with the observation of even antibiotic resistant mutants [31].

In the present study, only one case each of *Salmonella* and *Shigella* were detected in Gyeongsang-do and Gyeonggi-do, respectively. According to a study conducted by Adeyemi *et al*. about the microbial characteristics of leachate-contaminated groundwater, groundwater samples contaminated by simulated leachate had significantly high concentrations of *E*. *coli*, *Shigella* spp., and *Salmonella* spp. [32]. In a previous study in Busan, South Korea, 197 samples of drinking groundwater, which were not affected by leachate, were investigated [17]. The authors reported that *Salmonella* and *Shigella* were not detected anywhere [17]. In comparison with these previous studies, the groundwater tested in the present study had more potential for leachate contamination; however, either the leachate did not contain *Shigella* spp. or *Shigella* spp., or the bacteria were eliminated by interrupting factors before reaching the groundwater.

Choi *et al*. reported that 11.2% (22/197) of groundwater samples were contaminated with FC, and 36% (71/197) of the samples contained *E*. *coli* in 2005 in South Korea [17]. Comparing the present study with results from Choi *et al*., the water tested in the present study was less contaminated with *E*. *coli* (7.1%, 85/1,200), yet had higher frequency of FC (14.4%, 173/1,200). Thus, the groundwater near the carcass burial sites had more fecal contamination than other groundwater. 

NV was detected in six samples only in the spring, and it was not detected in the fall. According to Lee *et al*., the detection rate of NV in groundwater in South Korea was higher in the summer (June–August) (65/300 samples, 21.7%) than in the winter (October–December) (52/300 samples, 17.3%) [16]. The authors explained that the seasonal differences were due to the specific climate conditions and rainfall in the summer; accordingly, heavy rainfall leads to groundwater contamination from overflowing sewage or unfiltered wastewater [16]. Lee *et al*. examined a total of 1,090 groundwater samples, which were not exposed to leachate from burial sites and were treated by the groundwater treatment system for food-catering facilities. Samples were collected to evaluate the groundwater quality, and, among them, seven samples contained NV (0.64%, 7/1,090) [19]. This reported contamination rate of NV from groundwater is similar to our results, even though the samples in this present study were not collected near carcass burial sites. Taken together, these results indicate that the prevalence of NV from the groundwater collected in this study (0.5%, 6/1,200 samples) was not any higher than NV prevalence in other groundwater. 

Enterovirus was detected in 15 groundwater samples (1.3%, 15/1,200), and contaminated samples were mostly from Gyeonggi-do (2.1%, 12/584). Enterovirus could be a better indicator of water contamination than bacteria [33,34]; thus, this result implies that Gyeonggi-do has higher water contamination than the other provinces studied. The reason the Gyeonggi-do site contained more contaminated samples than other provinces is because of the high density population. Since many people live there, diverse contamination sources could be responsible for increased contamination, such as wastewater, sewage, and livestock wastewater. In a 2009 survey of the region, 5 of 29 (17%) groundwater samples were positive for enterovirus [18]. In comparison, these results show that the rate of enterovirus contamination in the present study (1.3%) was lower than that observed in the previous study [18], which examined groundwater not subjected to burial site leachate. 

To investigate the correlations within types of microorganisms and between physicochemical data (pH, temperature, turbidity, distance between burial site and groundwater, number of buried animals, and depth of the groundwater) and each microorganism, statistical analyses were performed; however, it was not possible to obtain reliable statistical results because the detection rates of pathogenic microorganisms were too low. Therefore, only the statistics associated with fecal coliform and *E*. *coli* were analyzed. However, reliable statistical data was not obtained (with the exception of the relationship between fecal coliform and *E*. *coli*); the *p*-values of all correlations between physicochemical data and microorganisms were not significantly different (*p* ≥ 0.05) (data not shown).

Overall, the detection of microorganisms was generally low, with the exception of *C*. *perfringens* and FC. Many factors affect the survival of pathogenic microorganisms from the leachate, including soil type, water table depth, rainfall, and soil permeability [8]. In addition, predation, filtration, and absorption of natural microbial populations effectively decrease the quantity of pathogens before they reach groundwater [8]. If the microorganisms reach the groundwater, fast water flow, grain size of the substrate, or pH of the water could kill pathogens [6].

Although the results of the present study indicate low possibility of contamination from burial site leachate, the possibility of contamination with leachate still remains, and concerns about the safety of the groundwater have arisen [8].

When using the burial method for disposal of carcasses, a strict protocol must be established to guarantee water safety. Before the burial, protocols for the selection of appropriate burial sites and for the processes of carcass sterilization and transport should be established. Additionally, after the burial, systematic management should be conducted with a focus on soil condition and viability of pathogens. Improper handling, unlawful burial, or inappropriate sterilization could lead to environmental contamination [35,36], whereupon, humans and animals could contract pathogenic diseases from the environment. Furthermore, depending on soil conditions, carcass decomposition could be delayed, and the period of time that leachate infiltrates the environment could be longer. In addition, inadequate disposal of carcasses could lead to failure in the purpose of eradicating pathogenic organisms. Kim *et al*. warned that inappropriate construction and poor selection of burial sites could lead to environmental contamination [9]. Furthermore, the decay period for a large number of animals commonly lasts at least 2 years [37], but humid soil, a high water table, and slow velocity of groundwater could cause the decomposition of carcasses to take more than ten years [38]. Moreover, according to Vinnerås *et al*., even under biomal conditions, *Salmonella* and an enveloped virus could still activate for one day, and *Bacillus cereus* spores were viable for up to 147 days (biomal—during incineration, concept of management for animal by-products (ABP); ABPs are stabilized with 2%–3% formic acid (85%, Addcon) that is used to inactivate pathogens) [39]. Furthermore, non-enveloped virus porcine parvovirus survived up to 168 days [39]. Considering these previous reports and our results, surveillance during and after the disposal process should be strictly managed with regard to pathogenic microorganisms. However, in South Korea, the burials were constructed without consideration of hydrogeology, and carcasses were hastily disposed in 2010 [9]. In addition, a proper systematic inspection has not been conducted in many regions where there is concern about groundwater contamination by burial leachate. Therefore, this study was necessary to monitor microbial safety of groundwater near the burials for ensuring health of the people who use the local groundwater. Furthermore, this report is valuable as the first nationwide study for investigation of pathogenic microorganisms in groundwater near carcass burial sites.
